# Evaluation of Less Invasive Sampling Tools for the Diagnosis of Cutaneous Leishmaniasis

**DOI:** 10.1093/ofid/ofae113

**Published:** 2024-02-28

**Authors:** Saskia van Henten, Mekibib Kassa, Helina Fikre, Roma Melkamu, Tigist Mekonnen, Dilargachew Dessie, Tadele Mulaw, Tadfe Bogale, Asinakew Engidaw, Arega Yeshanew, Lieselotte Cnops, Florian Vogt, Karel G M Moons, Johan van Griensven, Myrthe Pareyn

**Affiliations:** Department of Clinical Sciences, Institute of Tropical Medicine, Antwerp, Belgium; Leishmaniasis Research and Treatment Center, University of Gondar Hospital, Gondar, Ethiopia; Leishmaniasis Research and Treatment Center, University of Gondar Hospital, Gondar, Ethiopia; Leishmaniasis Research and Treatment Center, University of Gondar Hospital, Gondar, Ethiopia; Leishmaniasis Research and Treatment Center, University of Gondar Hospital, Gondar, Ethiopia; Leishmaniasis Research and Treatment Center, University of Gondar Hospital, Gondar, Ethiopia; Leishmaniasis Research and Treatment Center, University of Gondar Hospital, Gondar, Ethiopia; Leishmaniasis Research and Treatment Center, University of Gondar Hospital, Gondar, Ethiopia; Leishmaniasis Research and Treatment Center, University of Gondar Hospital, Gondar, Ethiopia; Leishmaniasis Research and Treatment Center, University of Gondar Hospital, Gondar, Ethiopia; Department of Clinical Sciences, Institute of Tropical Medicine, Antwerp, Belgium; Department of Clinical Sciences, Institute of Tropical Medicine, Antwerp, Belgium; National Centre for Epidemiology and Population Health, Research School of Population Health, College of Health and Medicine, Australian National University, Canberra, Australia Capital Territory, Australia; The Kirby Institute, University of New South Wales, Sydney, New South Wales, Australia; Julius Center for Health Sciences and Primary Care, University Medical Center Utrecht, Utrecht University, Utrecht, the Netherlands; Department of Clinical Sciences, Institute of Tropical Medicine, Antwerp, Belgium; Department of Clinical Sciences, Institute of Tropical Medicine, Antwerp, Belgium

**Keywords:** dental broach, diagnostics, Harpera microbiopsy, molecular biology, tape stripping discs

## Abstract

**Background:**

Diagnosis of cutaneous leishmaniasis (CL) usually relies on invasive samples, but it is unclear whether more patient-friendly tools are good alternatives for diverse lesions when used with polymerase chain reaction (PCR).

**Methods:**

Patients with suspected CL were enrolled consecutively in a prospective diagnostic accuracy study. We compared dental broach, tape disc, and microbiopsy samples with PCR as index tests, using PCR with skin slit samples as reference test. Subsequently, we constructed a composite reference test including microscopy, the 3 index tests and skin slit PCR, and we compared these same tests with the composite reference test. We assessed diagnostic accuracy parameters with 95% confidence intervals for all comparisons.

**Results:**

Among 344 included patients, 282 (82.0%) had CL diagnosed, and 62 (18.0%) CL absence, by skin slit PCR. The sensitivity and specificity by PCR were 89.0% (95% confidence interval, 84.8%–92.1%) and 58.1% (45.7%–69.5%), respectively, for dental broach, 96.1% (93.2%–97.8%) and 27.4% (17.9%–39.6%) for tape disc, and 74.8% (66.3%–81.7%) and 72.7% (51.8%–86.8%) for microbiopsy. Several reference test–negative patients were consistently positive with the index tests. Using the composite reference test, dental broach, and skin slit had similar diagnostic performance.

**Discussion:**

Dental broach seems a less invasive but similarly accurate alternative to skin slit for diagnosing CL when using PCR. Tape discs lack specificity and seem unsuitable for CL diagnosis without cutoff. Reference tests for CL are problematic, since using a single reference test is likely to miss true cases, while composite reference tests are often biased and impractical as they require multiple tests.

## Teaser

Dental broach seems a less painful but similarly accurate alternative to skin slit for diagnosing CL when using PCR for a wide variety of lesion presentations. Tape discs are easily contaminated and seem unsuitable for CL diagnosis without Ct-based cutoff.

## Graphical Abstract

**Graphical abstract:**
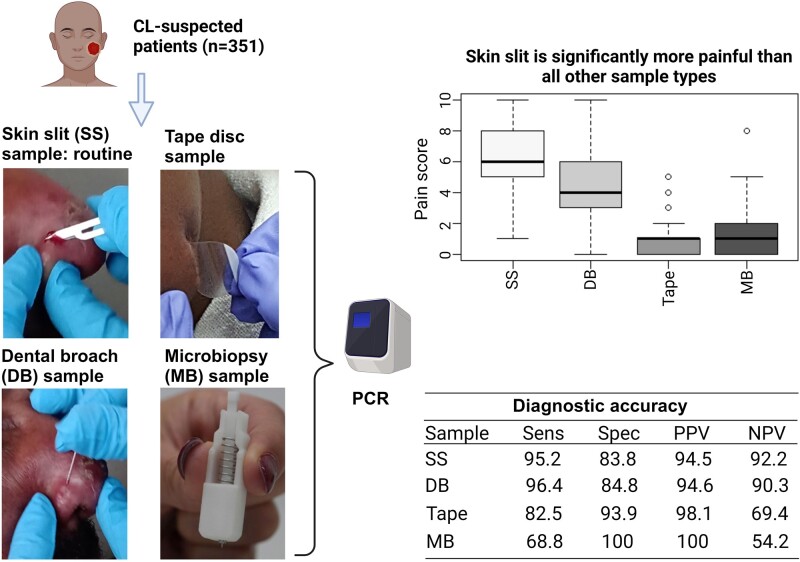
BioRender.com

Cutaneous leishmaniasis (CL) is an infectious skin condition caused by different species of *Leishmania* parasites. It is endemic in many countries, with an estimated yearly global incidence between 690 000 and 1 200 000 cases [1]. Owing to low sensitivity of commonly used diagnostic tools, such as microscopy or culture, there has been a shift toward molecular methods, most importantly polymerase chain reaction (PCR). Although diagnosis of CL is often still done on invasive samples, such as punch biopsy specimens and skin slit smears (Figure 1*D*), highly sensitive molecular techniques enable using more patient-friendly sampling devices.

**Figure 1.**
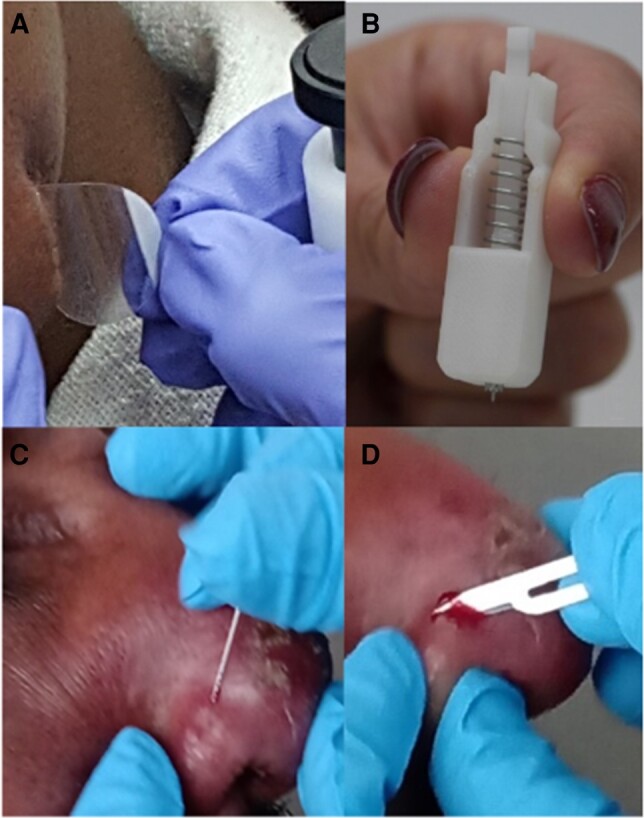
Sample collection tools. *A*, Tape disc samples are adhesive plastic circles 22 mm in size, supplied in sheets of 10 and stored in a pack with a total of 100 sheets. They strip away the topmost layer of the stratum corneum. *B*, Harpera microbiopsy devices contain a lancet that penetrates the skin to a depth of 350 µm, absorbing both blood and skin cell lysates, similar to the bite of a sandfly (Source: Tarl Prow). *C*, Dental broaches are sterile sample tools that are essentially small needles with barbed ends. The small barbs collect some tissue without major damage and allow for sample collection less contaminated with surface epithelium. *D*, Skin slit samples are taken after a small incision with a sterile scalpel, after which tissue is scraped from the inside of the skin.

In recent years, evidence has accumulated on the use of less invasive sampling methods, such as filter paper [2–5] and swab samples [6–10]. The majority of these studies were done on ulcerated lesions in Latin America. However, in other parts of the world, including Ethiopia, most lesions are dry, making these methods unsuitable since they still require either an incision or aspiration to obtain lesion tissue. Several other sample tools seem more suited for all lesion types.

Tape discs, which are coin sized and made of adhesive polyester (Figure 1*A*), are placed on the lesion and strip the top layer of the stratum corneum. They showed 100% sensitivity in Iran in microscopy-positive samples, but their performance in populations with suspected CL was not studied [11]. We reported promising results from a minimally invasive microbiopsy sampling tool (Figure 1*B*) [12], recently remarketed as Harpera (Trajan) in a small set of patients from Ethiopia. These results have not yet been substantiated in a larger patient group. Dental broaches (Figure 1*C*), small needles with barbed ends, are routinely supplied with the CL Detect rapid test (InBios), and older studies showed them to be promising [13, 14]. A few studies have provided information on these tools, but heterogeneity in terms of study design, reference tests, and small sample sizes make it difficult to assess and compare their value when used for molecular diagnosis. How painful each sampling method is for patients has never been assessed to our knowledge.

*Leishmania* parasites are unevenly distributed in CL lesions [15–17], and immune cell subsets were also shown to be spatially clustered in CL samples [18]. This complicates diagnostic studies since a negative result could occur even from positive patients by sampling from a lesion section with few parasites. This has led many studies to adopt a composite reference standard [6, 7, 19, 20] and place more emphasis on sensitivity when evaluating diagnostic tests.

In the current study, we compared the performance of tape disc, dental broach, and microbiopsy samples when tested with PCR (index tests) against skin slit PCR (reference test) for diagnosis of a variety of CL lesions in Ethiopia. We also determined the pain scores for these sampling types, and we explored different (composite) reference tests.

## METHODS

### Setting

This study took place at the Leishmaniasis Research and Treatment Center in Gondar University Hospital, Ethiopia. Routine diagnosis of CL uses a skin slit sample smeared on a slide, stained with Giemsa, and read under a microscope by 2 independent assessors, while PCR is used for research only.

### Design

A prospective cross-sectional diagnostic accuracy study was done to compare tape discs, dental broach, and microbiopsy samples analyzed by PCR as index tests, with skin slit analyzed by PCR as a reference test. We chose this reference test for the main analysis since our primary objective was to compare less invasive tests to the widely used skin slit. We used PCR because it can be used on all sample types. Alternative (composite) reference tests were explored (see Data Analysis). This study follows the Standards for Reporting of Diagnostic Accuracy (STARD) guidelines for reporting (Supplementary Table 1).

### Population and Recruitment

Patients with suspected CL were invited to participate consecutively from February 2019 until August 2022. Suspicion of CL was based on evaluation by treating physicians, usually in patients with a skin lesion of >2 weeks duration with erythema, crusts, plaques, ulceration, nodules, or papules in patients living in CL-endemic areas. Patients aged <2 years, those with lesions unsuitable for sample collection by skin slit or dental broach (eg, on the eyelid), and those with known CL diagnosis were excluded. Microbiopsy was only added to the study in August 2021, so results are available for a subset of patients.

### Consent and Ethical Considerations

This study was approved by the ethical review committees of the Institute of Tropical Medicine (1219/18), the University Hospital of Antwerp (18/08/085), and the University of Gondar (O/V/P/RCS/05/626/2019). Written informed consent was collected from study participants or from the guardian for minors. Assent was also requested from patients aged 12–17 years. The study is registered at ClinicalTrials.gov (NCT03837431). Patients specifically gave consent for the taking and use of photographs, as long as they could not be recognized.

### Clinical Data Collection

The study clinicians measured the index lesion's largest diameter, counted the number of active lesions, classified patients as having localized, mucocutaneous, or diffuse CL, as described elsewhere [21], and noted whether particular lesion features were present.

### Skin Sample Collection

Trained laboratory technicians chose the lesion and sampling site, aiming for the most active site (usually the border of the lesion). Samples were collected from the same location as much as possible, starting with tape (least invasive), followed by the more invasive sample devices (microbiopsy, dental broach and skin slit). Topical EMLA cream (5% lidocaine-prilocaine; AstraZeneca) was applied for 30–60 minutes before collection of the dental broach and skin slit to limit patient discomfort. Until June 2021, skin slit and dental broach samples were stored in CL Detect kit lysis buffer; after that, all samples were stored in 300 µL of 1X DNA/RNA Shield (Zymo Research). Patients were asked to rate the pain for each sample method, on a scale from 0 to 10.

After cleaning with sterile saline, tape disc samples (Figure 1*A*; Monaderm) were pressed on the lesion for 10 seconds using the company-supplied plunger, which was cleaned with alcohol (and also bleach after June 2021) between patients. Tapes were folded inward and placed into collection tubes.

Harpera microbiopsy devices (Trajan; Figure 1*B*) were applied on the lesion for 10 seconds. Samples were flushed from the lancet and then stored after the lancet was discarded. Dental broaches (Figure 1*C*) were inserted in the lesion up to 1 cm, rotated several times, and pulled back quickly for retrieval. Sample tissue was flushed off, and the dental broach was discarded.

Skin slits were collected using a small incision, from which tissue was scaped from the inner wound surface with the blade at a perpendicular angle. Two skin slits (Figure 1*D*) were taken from the same site. One was processed for microscopy according to routine; for the other, the tissue was flushed from the scalpel and stored. All samples were frozen at −80°C.

### DNA Extraction

DNA was extracted using the Maxwell 16 LEV Blood DNA kit (Promega) and the automated Maxwell 16 device (AS1000; Promega) per the manufacturer's instructions. In each extraction batch of 15 samples, a negative extraction control (NEC; lysis buffer only) was included.

### PCR Protocols

*Leishmania* DNA was detected using LC kinetoplast DNA (kDNA) PCR, real-time PCR targeting kDNA, with amplification up to 50 cycles, as described elsewhere [22], using the Rotor-Gene Q instrument (Qiagen). Each run contained 2 positive and negative PCR controls; NECs were included in duplo. Results were expressed in cycle threshold (Ct) values. Results were validated as shown in Supplementary Figure 1. PCR analyses were performed in batch, and interpretation was done blinded to the other test results and clinical information.

### Data Analysis

Data were collected on paper and entered into a Kobo Toolbox [23] database. Analysis was done using R software, version 4.1.3. Numbers, proportions, medians, and interquartile ranges (IQRs) were used to describe patients. PCR results (for both index and reference tests) were categorized as positive, negative, invalid, and undetermined (Supplementary Figure 1). Patients with an undetermined reference test result (insufficient skin material) were excluded from the diagnostic accuracy analysis (as their “true” disease status could not be determined), whereas undetermined results for the index tests were included in the analysis. Invalid results (for both index and reference tests) due to problems with the NEC were primarily grouped as negative, with secondary analyses grouping them as positive or removing them. Sensitivity, specificity, and positive and negative predictive values with 95% confidence intervals (CIs) were calculated for the 3 index tests (dental broach, tape, and microbiopsy with PCR), against the reference (skin slit PCR).

We explored microscopy as reference in addition to a composite reference test, because we expected skin slit PCR to be an imperfect reference and we wanted to compare skin slit with the other sample types. A composite reference test including skin slit, tape, dental broach, and microbiopsy PCR together with skin slit microscopy was constructed, which was a positive if ≥2 test results were positive. Mann-Whitney tests were used to compare pain scores and Ct values, χ^2^ and Fisher exact tests to compare sensitivities and specificities, and Spearman rank correlation coefficients to assess correlation between Ct values for the different sample types. Receiver operating characteristic curves were used to explore optimal cutoffs for Ct values, by imputing negative PCR results with a Ct of 50 and with the aim to achieve the highest sum of sensitivity and specificity.

### Sample Size

The sample size of 350 was calculated based on objectives described in [21], but the primary objective changed; hence, we calculated the widths of the 95% CIs widths for the sensitivity and specificity of the tape disc samples. Based on an overall reference test positivity of 80% [21], an estimated sensitivity of 90% and specificity of 50% for the tape disc samples, the calculated 95% CI was between 86% and 93% for sensitivity and between 41% and 60% for specificity.

## RESULTS

Among 484 patients screened, 351 are described in this article, with 7 patients having insufficient material for the reference test (Supplementary Figure 2). Because microbiopsy was added to the study later, its results are available for only 148 patients. Patient characteristics (Supplementary Table 2) are similar to those previously described [21], with patients having severe, diverse, and long-standing lesions. About half of all patients (168 [47.9%]) were microscopy positive.

### Reference and Index Test Positivity Rates

**Figure 2.**
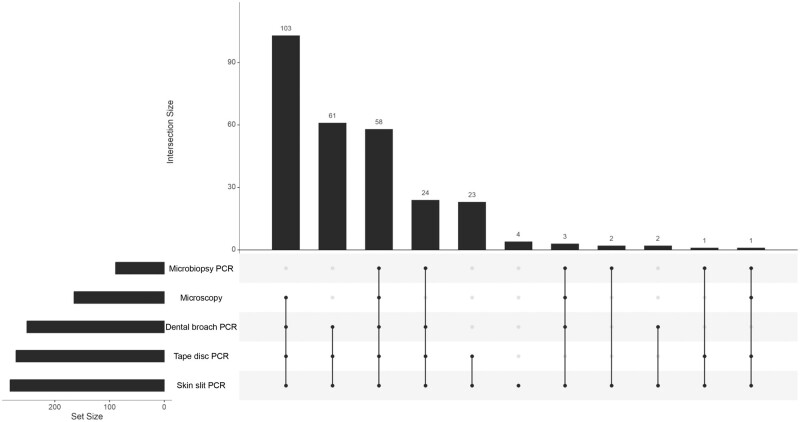
Upset plot of the different tests used, showing overlapping test results for patients with suspected cutaneous leishmaniasis testing positive with skin slit polymerase chain reaction (PCR) as the reference test. The intersection size shows the number of patients positive for a certain selection of tests, which is indicated below the respective bars. The total number of patients positive for each test is shown by the horizontal bars.

An overview of index and reference test results is shown in Supplementary Figure 2, and the overlap between results in Figure 2. Overall, positivity rates were highest for tape disc samples (321 of 351 [91.5%]), followed by skin slit (282 of 351 [80.3%]), and dental broach (281 of 351 [80.1%]) and lowest for microbiopsy (100 of 148 [67.6%])., Fifty-three skin slit results (15.1%) were negative, 9 (2.6%) invalid, and 7 (2.0%) undetermined. Invalid and undetermined results were seen in low numbers in all sample types.

### Diagnostic Accuracy of Index Tests

Skin slit results as reference test were available for 344 patients. Grouping the invalid reference test results as negative, 282 (82.0%) patients were case patients, and 62 (18.0%) were non–case patients. Diagnostic accuracy of the 3 index tests is shown in Table 1. The positive predictive value was >85% for all index tests. PCR with dental broach samples had a sensitivity of 89.0% (95% CI, 84.8%–92.1%) and specificity of 58.1% (45.7–69.5). PCR with tape disc samples had very high sensitivity (96.1% [95% CI, 93.2%–97.8%]), but specificity was poor, at 27.4% (17.9%–39.6%). Microbiopsy had a sensitivity of 74.8% (95% CI, 66.3%–81.7%), with a specificity of 72.7%. Grouping invalid results as positive (not shown) or missing (Supplementary Table 3) did not significantly change diagnostic performance.

**Table 1.** Diagnostic Accuracy of Index Tests Compared With Polymerase Chain Reaction on Skin Slit as Reference Test

Test	Case Patients (n = 282)		Non–Case Patients (n = 62)^a^		Diagnostic Accuracy (95% CI), %			
	Pos	Neg	Pos	Neg	Sensitivity	Specificity	PPV	NPV
DB	251	31	25	36	89.0 (84.8–92.1)	58.1^b^ (45.7–69.5)	90.9 (87.0–93.8)	53.7 (41.9–65.1)
TD	271	8	43	17	96.1^c^ (93.2–97.8)	27.4^c^ (17.9–39.6)	86.3 (82.1–89.7)	68.0 (48.4–82.8)
MB	89	29	6	16	74.8^d^ (66.3–81.7)	72.7^d^ (51.8–86.8)	93.7 (86.9–97.1)	35.6 (23.2–50.2)

Abbreviations: CI, confidence interval; DB, polymerace chain reaction (PCR) on dental broach samples; MB, PCR on microbiopsy samples; NPV, negative predictive value; PPV, positive predictive value; TD, PCR on tape disc samples.

^a^Non–case patients include 9 patients with invalid reference test results, which were primarily analyzed as negative.

^b^Specificity for DB was calculated using 62 as the denominator, even though 1 DB result from a non–case patient was undetermined.

^c^For TD, sensitivity was calculated using 282 as the denominator, even though 3 case patients had undetermined TD results, and specificity was calculated using 62 as the denominator, even though 2 non–case patients had undetermined TD results.

^d^For MB, sensitivity was calculated using 119 as the denominator, even though 1 case patient had an undetermined MB result, and specificity was calculated using 22 as the denominator. Because MB was introduced later in the study (August 2021), results include only a subset of patients.

### Skin Slit PCR as an Imperfect Reference Test

**Figure 3.**
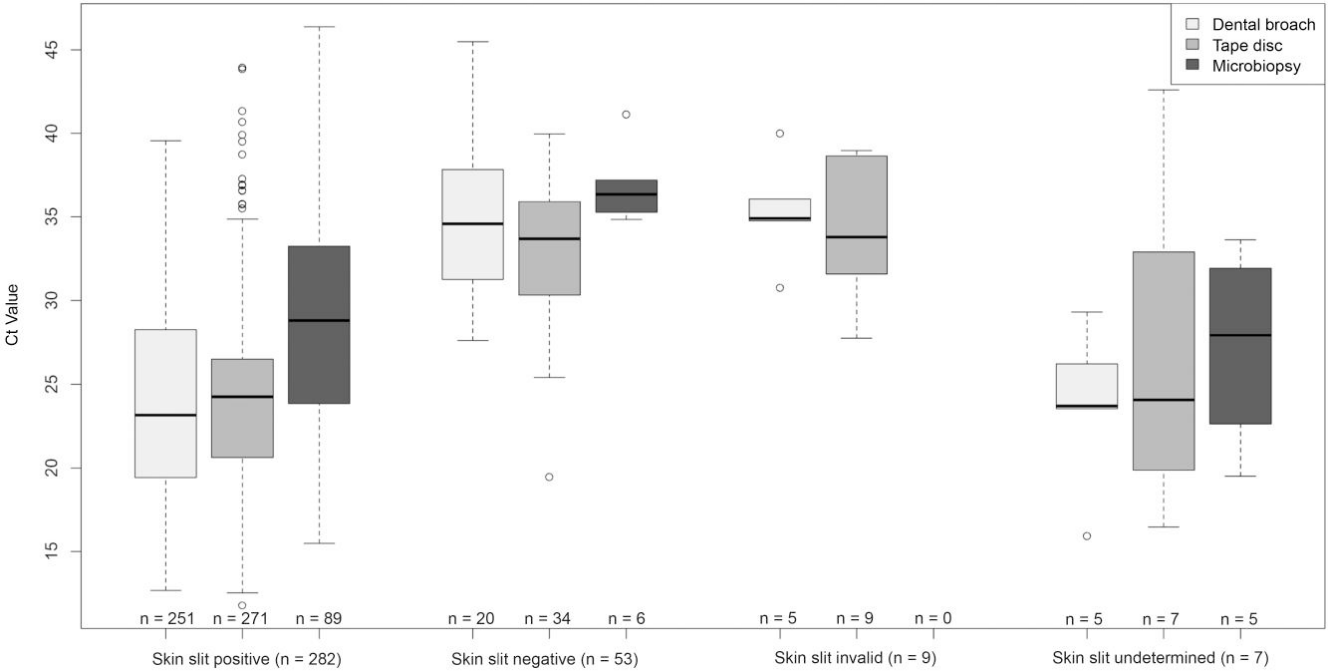
Results of dental broach, tape disc and microbiopsy stratified by skin slit polymerase chain reaction (PCR) result. Cycle threshold (Ct) values are plotted in light gray for dental broach–positive, in medium gray for tape disc–positive, and in dark gray for microbiopsy-positive samples. The number of positive samples is indicated below each bar. The Ct values for PCR with the other sample types for patients who were skin slit negative or invalid were significantly higher than the values for skin slit–positive patients. Patients with skin slit­–undetermined results sometimes had low Ct values for the other sample types. Many skin slit–negative results were positive for the other sample types.

Figure 3 shows the number of positive results and their respective Ct values for the index tests, stratified by the skin slit result as reference test. Ct values of dental broach, tape disc, and microbiopsy results for reference test–positive patients were significantly lower than for reference test–negative patients (*P* < .001 for dental broach and tape and *P* = .003 for microbiopsy; Mann-Whitney test), with a median Ct value of about 35. However, many of the 53 patients who were skin slit PCR negative were positive for the index tests. Two reference test–negative patients (3.8%) were positive for all index tests, 17 (32.0%) for ≥2 index tests, and 20 (37.7%) for a single index test. Only 14 patients were consistently negative for both index and reference tests.

### Pain Scores

Pain scores for the different sample types are shown in Supplementary Figure 3. Skin slit was significantly more painful (*P* < .001; Mann-Whitney test) than all other sample methods, with a median pain score of 6 (IQR, 5–8). Dental broach had a pain score of 4 (IQR, 3–6) higher (*P* < .001) than scores for both tape and microbiopsy, which both had a median pain score of 1 (IQRs, 0–1 and 0–2, respectively).

### Correlation of Ct Values

Scatterplots comparing Ct values of the different samples tested by PCR are shown in Supplementary Figure 4. Correlation was strongest between skin slit and dental broach (ρ = 0.82; Spearman), lower for skin slit and microbiopsy (ρ = 0.65; Spearman) and weakest between tape and skin slit. Data showed that patients can have large differences in Ct values between samples, which seemed random and in both directions for comparisons between skin slit, tape, and dental broach samples. Ct values were systematically higher for microbiopsy than for other sample types, which is highlighted in Figure 3.

### Contamination

In 90 NECs, 41 (45.6%) had a Ct value in ≥1 of the duplicates. Mostly (in 28 of 41 [68.3%]), this was due to cross-contamination (only 1 of 2 duplicates from the same NEC positive), while for 13 (31.7%) this seemed to occur during extraction (NEC duplicates repeatedly positive). The median Ct value of NECs (taking lowest Cts) with cross-contamination was 39.8 (IQR, 34.3–43.8); this value was 36.2 (34.940.4) for samples with contamination during extraction.

### Interpretation of Index Test Results With High Ct Values

Because several patients had multiple positive samples with a Ct value >35, we used receiver operating characteristic curves to explore at which cutoff sensitivity and specificity were maximized. These cutoffs were 33.5 for dental broach, 27.3 for tape, and 34.8 for microbiopsy PCR, using skin slit PCR as the reference.

### Alternative Reference Tests and Using a Cutoff for Ct Values

With microscopy as the reference test or with the composite reference test (Supplementary Table 4), diagnostic performances of skin slit and dental broach PCR were very similar. Using microscopy as the reference, the sensitivity of all tests was good, but the specificity was poor. We also constructed an adapted composite reference that was considered positive either if the result of microscopy was positive or ≥2 PCR results were positive with a Ct value of <34.3 (based on the 95% CI for the Ct values of cross-contamination) and <27 for tape, since contamination seemed to happen at lower Ct values for tape discs. With this reference, there were 252 case patients and 99 non–case patients.

Owing to contamination, we used the same cutoffs described above for the index tests, to decrease the chance of false-positives. Diagnostic accuracy is shown in Table 2 (sensitivity analysis to explore incorporation bias in Supplementary Table 5). Sensitivity for skin slit and dental broach samples was >95%, whereas specificity was about 85%. Tape sensitivity was 82.5%, with specificity of 93.9%. Using cutoffs lowers the sensitivity of microbiopsy to <70%, while specificity is 100%.

**Table 2.** Diagnostic Accuracy Using Cycle Threshold Value Cutoffs and a Composite Reference Test

Test	Case Patients (n = 252)		Non–Case Patients (n = 99)		Diagnostic Accuracy (95% CI), %			
	Pos	Neg	Pos	Neg	Sensitivity^a^	Specificity^b^	PPV	NPV
SS	240	7	14	83	95.2 (91.9–97.3)	83.8 (75.3–89.8)	94.5 (91.0–96.7)	92.2 (84.8–96.2)
DB	243	9	14	84	96.4 (93.4–98.1)	84.8 (76.5–90.6)	94.6 (91.1–96.7)	90.3 (82.6–94.8)
TD	208	41	4	93	82.5 (77.4–86.7)	93.9 (87.4–97.2)	98.1 (95.2–99.3)	69.4 (61.2–76.6)
MB^c^	75	33	0	39	68.8 (59.6–76.7)	100 (91.0–100)	100 (95.1–100)	54.2 (42.7–65.2)

Abbreviations: CI, confidence interval; DB, polymerase chain reaction (PCR ) on dental broach samples; MB, PCR on microbiopsy samples; NPV, negative predictive value; PPV, positive predictive value; SS, skin slit samples with PCR; TD, PCR on tape disc samples.

^a^Sensitivity was calculated using 252 as the denominator for SS, DB, and TD, even though 5 SS and 3 TD results in case patients were undetermined.

^b^Specificity was calculated using 99 as the denominator, even though 2 SS, 1 DB, and 2 TD results in non–case patients were undetermined.

^c^The denominators for MB calculations were 109 case and 39 non–case patients, even though 1 case patient had an undetermined MB result. Because MB was introduced later in the study (August 2021), results include only a subset of patients.

## DISCUSSION

The dental broach seems to be a good alternative to skin slit collection, as it has good diagnostic performance, is inexpensive, and is less painful. Positivity rates were similar, Ct values were correlated, and good concordance was seen between most patients, with discrepancies in negative results likely caused by parasite distribution and inconsistent sample collection. Dental broach was previously studied in combination with the CL Detect Rapid test, which showed that using the rapid test on a dental broach had lower sensitivity than skin slit [21, 24]. Dental broach was also shown to be promising as a sampling tool for loop-mediated isothermal amplification, where it reached a sensitivity of 94%, with PCR on dental broach in combination with skin slit microscopy as a reference test [19]. Using dental broach for microscopy has also been done [13, 25], and was actually reported to have higher or similar positivity rates compared with microscopy on skin slit [14, 26].

Results from the microbiopsy were less promising than in our pilot study, where all 29 skin slit PCR positives were confirmed with microbiopsy PCR [12].This difference could be due to changes in sample processing: because retrieving the microbiopsy lancet was difficult, the sample was flushed out instead. In addition, more stringent criteria were used to call samples positive. Based on the diagnostic accuracy reported here and the current high cost of microbiopsy (10–15 euros), its use for routine diagnosis of CL seems limited, although recent design innovations that should simplify lancet retrieval may improve performance. Still, it remains promising as a research tool to measure treatment outcomes or transmission, especially since sample collection appears more reproducible than other tools.

PCR contamination was frequently observed, despite extensive cleaning. Most contamination seemed to occur not during extraction or sample collection, but during PCR preparation. This could be related to the small tubes of the Rotorgene Q, the highly sensitive kDNA target, and the high proportion of positive samples.

Specificity was especially low for tape discs, probably caused by contamination before or during sample collection from one patient to the next, because tapes are not provided in sterile packaging. Even though tapes are patient friendly, inexpensive, and easy to use, we do not currently advise using them for CL diagnosis, although using a stringent Ct cutoff at about 27 may be a solution. Extra measures should be taken to limit contamination by using a single sheet (10 tapes) for only one patient, storing unused tapes in closed containers, and thoroughly cleaning the pressure device with bleach and alcohol before and after each use. Nevertheless, tapes hold promise because of their low cost and because they require little skill for collection. As such, they are suitable for decentralized sample collection with regular shipment to referral laboratories, as they can be stored at room temperature for up to a month if placed in preservatives [27].

Contamination further complicates the task of setting a suitable reference test. Other groups have noted this problem of imperfect reference tests that fail to detect all positive cases owing to variation in sample collection and heterogeneity in parasite distribution [6, 19]. Especially in patients with low parasite loads, this can lead to discordant results. Therefore, some researchers resorted to using a composite reference test that incorporates the index tests to determine true cases [2, 3, 10]. Although this can increase specificity, it comes with incorporation bias as an important methodological issue. We show that choosing an appropriate reference test for CL diagnostic studies is crucial, as it highly affects the reported diagnostic accuracy findings, especially for specificity estimates. In the current study, we primarily used skin slit PCR as a reference, as skin slit is the routinely used sampling method, and we wanted to compare diagnostic performance of dental broach, tape disc, and microbiopsy using the same test method. However, for future studies, we recommend using a composite reference that includes ≥2 moderately sensitive and specific tests that do not include the index tests under study to optimize sensitivity. In our case, this would mean microscopy positive or ≥2 PCR results that are clearly positive with Ct values below the levels seen in contaminated samples.

The low specificity that was observed when comparing dental broach and tape with skin slit as the reference could either be “false-positives”—true non-CL cases occurring owing to PCR contamination—or could occur because the reference test fails to detect all cases picked up in some other sample types. Because contamination occurred frequently in our setup, we used a cutoff to increase specificity for the index tests, which is important for a nondeadly disease like CL, in which overtreatment should be avoided. The cutoffs we chose were based on the Ct values of the observed cross-contamination but were also similar to the optimal cutoff Ct value, which maximized the combined sensitivity and specificity compared with skin slit PCR. For simplicity, we generally recommend using a cutoff Ct value of 35 to call a PCR result positive for dental broach, skin slit, and microbiopsy and an even lower cutoff of 27 for tape discs, to avoid overtreatment.

Weaknesses of the current study included frequent occurrence of contamination, which complicates interpretation of results, and a low number of non-CL cases, thereby limiting the precision of our specificity estimates. Because we wanted to study diagnostic performance in our population of interest (suspected CL), we did not include nonendemic controls. Bias due to inconsistent sampling is likely to have occurred, related to the operator, the device, and the lesion. Whereas microbiopsies are relatively operator independent, we observed that tape disc samples retrieved more tissue in lesions with crusts, dental broach sample collection seemed easier in ulcerated lesions, and skin slit collection was more difficult in sites that bleed easily. Pain scores for skin slit and dental broach were collected after application of topical lidocaine, but tape disc and microbiopsy were taken before, since lidocaine potentially interfered with immunological techniques planned for a substudy on the tapes. As such, we likely underestimated the true difference in pain scores between dental broach/skin slit (which would have been higher without lidocaine application) and microbiopsy/tape disc samples.

Although we show that dental broach samples could be a more patient-friendly alternative to skin slit samples when testing with PCR, practical uptake in low-resource settings is complicated by difficulties in procuring PCR reagents and the high costs of molecular techniques. Strengths of our study are a large sample size, including a wide variety of clinical presentations, and rigorous evaluation of PCR results with extensive use of controls, which is often overlooked when using the highly sensitive kDNA target.

In conclusion, we show that dental broach samples are very similar to skin slit samples for the diagnosis of CL when used with PCR, and this seems a more patient-friendly alternative for sample collection. We recommend its use combined with PCR in settings with routine molecular testing, and we also recommend further exploration as a routine sampling tool in combination with microscopy testing in resource-limited settings.

## Supplementary Material

ofae113_Supplementary_Data

## Supplementary Data

Supplementary materials are available at the *Open Forum Infectious Diseases* online. Consisting of data provided by the authors to benefit the reader, the posted materials are not copyedited and are the sole responsibility of the authors, so questions or comments should be addressed to the corresponding author.

## References

[1] Alvar J, Vélez ID, Bern C, et al Leishmaniasis worldwide and global estimates of its incidence. PLoS One 2012; 7:e35671. 22693548 10.1371/journal.pone.0035671 PMC3365071

[2] Al-Jawabreh A, Dumaidi K, Ereqat S, et al A comparison of the efficiency of three sampling methods for use in the molecular and conventional diagnosis of cutaneous leishmaniasis. Acta Trop 2018; 182:173–7. 29522706 10.1016/j.actatropica.2018.03.001

[3] Boggild AK, Valencia BM, Espinosa D, et al Detection and species identification of *Leishmania* DNA from filter paper lesion impressions for patients with American cutaneous leishmaniasis. Clin Infect Dis 2010; 50:e1–6. 19947858 10.1086/648730

[4] Kato H, Cáceres AG, Mimori T, et al Use of FTA cards for direct sampling of patients’ lesions in the ecological study of cutaneous leishmaniasis. J Clin Microbiol 2010; 48:3661–5. 20720027 10.1128/JCM.00498-10 PMC2953078

[5] Miranda A, Saldaña A, González K, et al Evaluation of PCR for cutaneous leishmaniasis diagnosis and species identification using filter paper samples in Panama, Central America. Trans R Soc Trop Med Hyg 2012; 106:544–8. 22818741 10.1016/j.trstmh.2012.05.005

[6] Adams ER, Gomez MA, Scheske L, et al Sensitive diagnosis of cutaneous leishmaniasis by lesion swab sampling coupled to qPCR. Parasitology 2014; 141:1891–7. 25111885 10.1017/S0031182014001280 PMC4654403

[7] Blaizot R, Simon S, Ginouves M, et al Validation of swab sampling and SYBR green-based real-time PCR for the diagnosis of cutaneous leishmaniasis in French Guiana. J Clin Microbiol 2021; 59:e02218-20. 33148706 10.1128/JCM.02218-20 PMC8111157

[8] Boggild AK, Valencia BM, Veland N, et al Non-invasive cytology brush PCR diagnostic testing in mucosal leishmaniasis: superior performance to conventional biopsy with histopathology. PLoS One 2011; 6:e26395. 22046280 10.1371/journal.pone.0026395 PMC3203107

[9] Daoui O, Ait Kbaich M, Mhaidi I, et al The role of sampling by cotton swab in the molecular diagnosis of cutaneous leishmaniasis. Transbound Emerg Dis 2021; 68:2287–94. 33094519 10.1111/tbed.13886

[10] Valencia BM, Veland N, Alba M, et al Non-invasive cytology brush PCR for the diagnosis and causative species identification of American cutaneous leishmaniasis in Peru. PLoS One 2012; 7:e49738. 23185421 10.1371/journal.pone.0049738 PMC3504088

[11] Taslimi Y, Sadeghipour P, Habibzadeh S, et al A novel non-invasive diagnostic sampling technique for cutaneous leishmaniasis. PLoS Negl Trop Dis 2017; 11:e0005750. 28704463 10.1371/journal.pntd.0005750 PMC5526608

[12] Churiso G, van Henten S, Cnops L, et al Minimally-invasive microbiopsies as an improved sampling method for the diagnosis of cutaneous leishmaniasis. Open Forum Infect Dis 2020; 7:ofaa364. 32939358 10.1093/ofid/ofaa364 PMC7486950

[13] Griffiths WA, Dutz W. Repeated tissue sampling with a dental broach: a trial in cutaneous leishmaniasis. Br J Dermatol 1975; 93:43–5. 1103933 10.1111/j.1365-2133.1975.tb06474.x

[14] Sharquie KE, Hassen AS, Hassan SA, Al-Hamami IA. Evaluation of diagnosis of cutaneous leishmaniasis by direct smear, culture and histopathology. Saudi Med J 2002; 23:925–8. 12235464

[15] Thomaz C, de Mello CX, Espíndola OM, et al Comparison of parasite load by qPCR and histopathological changes of inner and outer edge of ulcerated cutaneous lesions of cutaneous leishmaniasis. PLoS One 2021; 16:e0243978. 33476320 10.1371/journal.pone.0243978 PMC7819606

[16] Suárez M, Valencia BM, Jara M, et al Quantification of *Leishmania* (*Viannia*) kinetoplast DNA in ulcers of cutaneous leishmaniasis reveals inter-site and intersampling variability in parasite load. PLoS Negl Trop Dis 2015; 9:e0003936. 26204525 10.1371/journal.pntd.0003936 PMC4512720

[17] Sevilha-Santos L, Dos Santos Júnior ACM, Medeiros-Silva V, et al Accuracy of qPCR for quantifying *Leishmania* kDNA in different skin layers of patients with American tegumentary leishmaniasis. Clin Microbiol Infect 2019; 25:242–7. 29730222 10.1016/j.cmi.2018.04.025

[18] Dey NS, Senaratne S, Somaratne V, et al Early reduction in PD-L1 expression predicts faster treatment response in human cutaneous leishmaniasis. J Clin Invest 2021; 131:e142765. 34609968 10.1172/JCI142765 PMC8592550

[19] Vink MMT, Nahzat SM, Rahimi H, et al Evaluation of point-of-care tests for cutaneous leishmaniasis diagnosis in Kabul, Afghanistan. EBioMedicine 2018; 37:453–60. 30396855 10.1016/j.ebiom.2018.10.063 PMC6286266

[20] Gomes CM, Cesetti V, De PA, et al Field validation of SYBR green- and TaqMan-based real-time PCR using biopsy and swab samples to diagnose American tegumentary leishmaniasis in an area where *Leishmania* (*Viannia*) *braziliensis* is endemic. J Clin Microbiol 2017; 55:526–34. 27927916 10.1128/JCM.01954-16 PMC5277523

[21] van Henten S, Fikre H, Melkamu R, et al Evaluation of the CL Detect Rapid Test in Ethiopian patients suspected for cutaneous leishmaniasis. PLoS Negl Trop Dis 2022; 16:e0010143. 35041672 10.1371/journal.pntd.0010143 PMC8797207

[22] Merdekios B, Pareyn M, Tadesse D, et al Evaluation of conventional and four real-time PCR methods for the detection of *Leishmania* on field-collected samples in Ethiopia. PLoS Negl Trop Dis 2021; 15:e0008903. 33434190 10.1371/journal.pntd.0008903 PMC7802924

[23] KoboToolbox . Available at: https://www.kobotoolbox.org/.

[24] Grogl M, Joya CA, Saenz M, et al Evaluation of a diagnostic device, CL Detect rapid test for the diagnosis of new world cutaneous leishmaniasis in Peru. PLoS Negl Trop Dis 2023; 17:e0011054. 36913433 10.1371/journal.pntd.0011054 PMC10010545

[25] Al-Heany AR, Sharquie KE, Al-Najar SA, Prof A, Noaimi AA. Cutaneous leishmaniasis: comparative techniques for diagnosis. IOSR J Dent Med Sci 2014; 13:33–7.

[26] ul Bari A, Azam S, Mahmood T. Comparison of various cytodiagnostic tests in the rapid diagnosis of cutaneous leishmaniasis. J Pak Assoc Dermatol 2010; 20:63–9.

[27] Zymo Research. DNA/RNA Shield . Available at: https://zymoresearch.eu/collections/dna-rnashield/products/dna-rna-shield. Accessed 1 January 2024.

