# Supplementary material for: Evaluation of Less Invasive Sampling Tools for the Diagnosis of Cutaneous Leishmaniasis
Source: Open Forum Infect Dis. 2024 Feb 28;11(4):ofae113. doi: 10.1093/ofid/ofae113 (PMC10977625; doi:10.1093/ofid/ofae113)
Supplement: ofae113_Supplementary_Data [file ofae113_supplementary_data.zip › 12. Supplementary Figure1.docx]

**
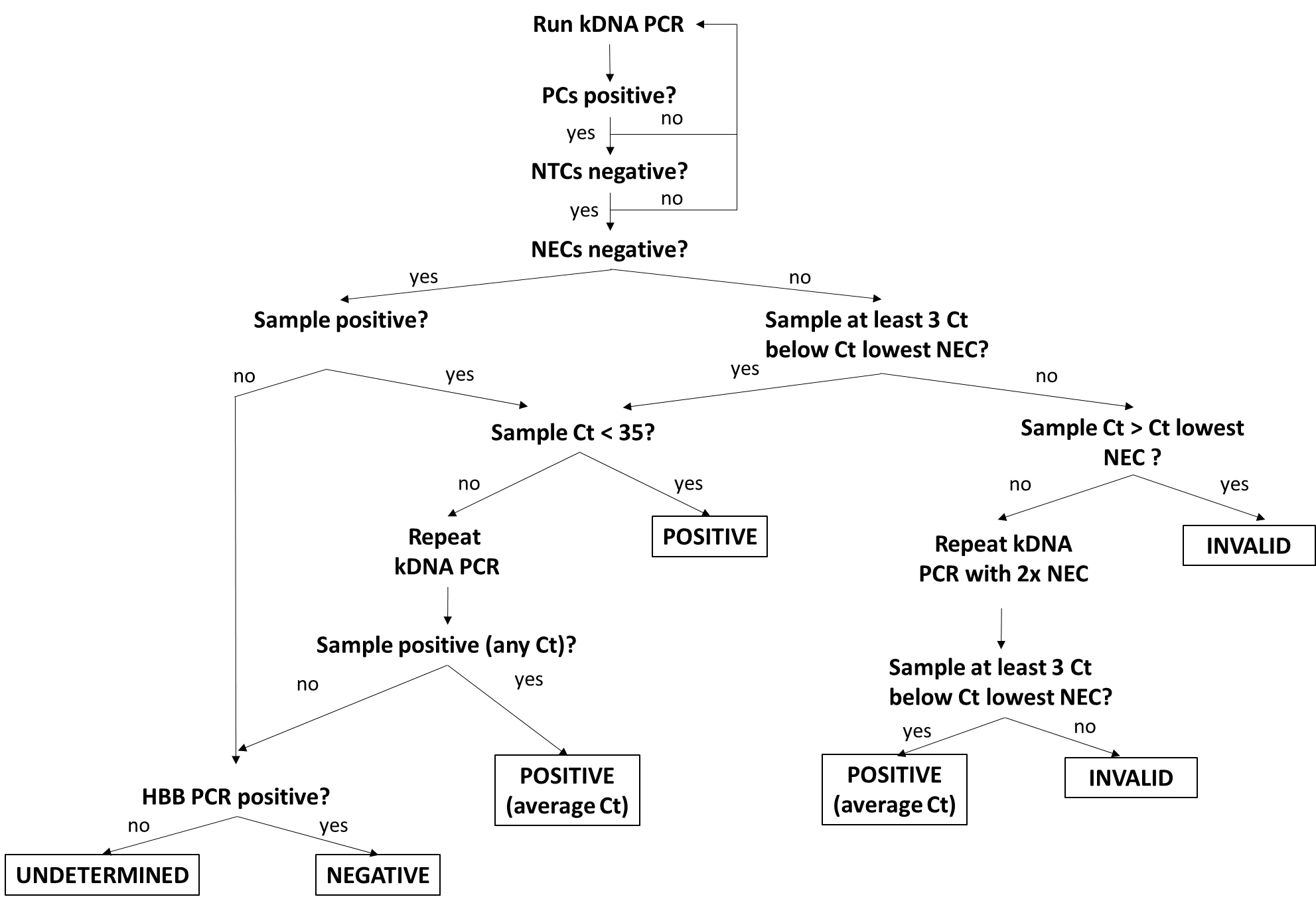
**

**Supplementary Figure 1: Validation and interpretation of PCR results** PC: positive control, NTC: negative template control, NEC: negative extraction control, HBB: human beta globin gene. *Leishmania* DNA was detected by a real-time PCR targeting kinetoplast DNA (kDNA) with amplification up to 50 cycles as previously described [22], using the Rotor-Gene Q instrument (Qiagen, Venlo, The Netherlands). Each run contained two positive and negative PCR controls and the NECs for each extraction batch were included *in duplo*. Results were expressed in cycle threshold (Ct)-values. Samples were called positive for Ct-values under 35 and for Ct-values >35 if they were positive in the repeat as well. Samples that were negative in the repeat were called negative. If any of the two NECs for a particular extraction batch were positive (any Ct), only samples with a Ct-value at least three Ct-values lower than the lowest NEC Ct were considered positive, otherwise the sample was invalid (as contamination cannot be excluded). Samples that were negative for kDNA were tested for PCR targeting the human beta globin gene (HBB) to monitor extraction efficiency and PCR inhibition [30]; samples with a negative HBB PCR (Ct >35) were classified as undetermined (as this can indicates insufficient sample material or PCR inhibition). Mean Ct-values were computed for samples for which several repeat runs were done.
