# Supplementary material for: Evaluation of Less Invasive Sampling Tools for the Diagnosis of Cutaneous Leishmaniasis
Source: Open Forum Infect Dis. 2024 Feb 28;11(4):ofae113. doi: 10.1093/ofid/ofae113 (PMC10977625; doi:10.1093/ofid/ofae113)

**Supplementary Figure 2. Overview of included patients and index and reference test results using skin slit PCR as reference test**


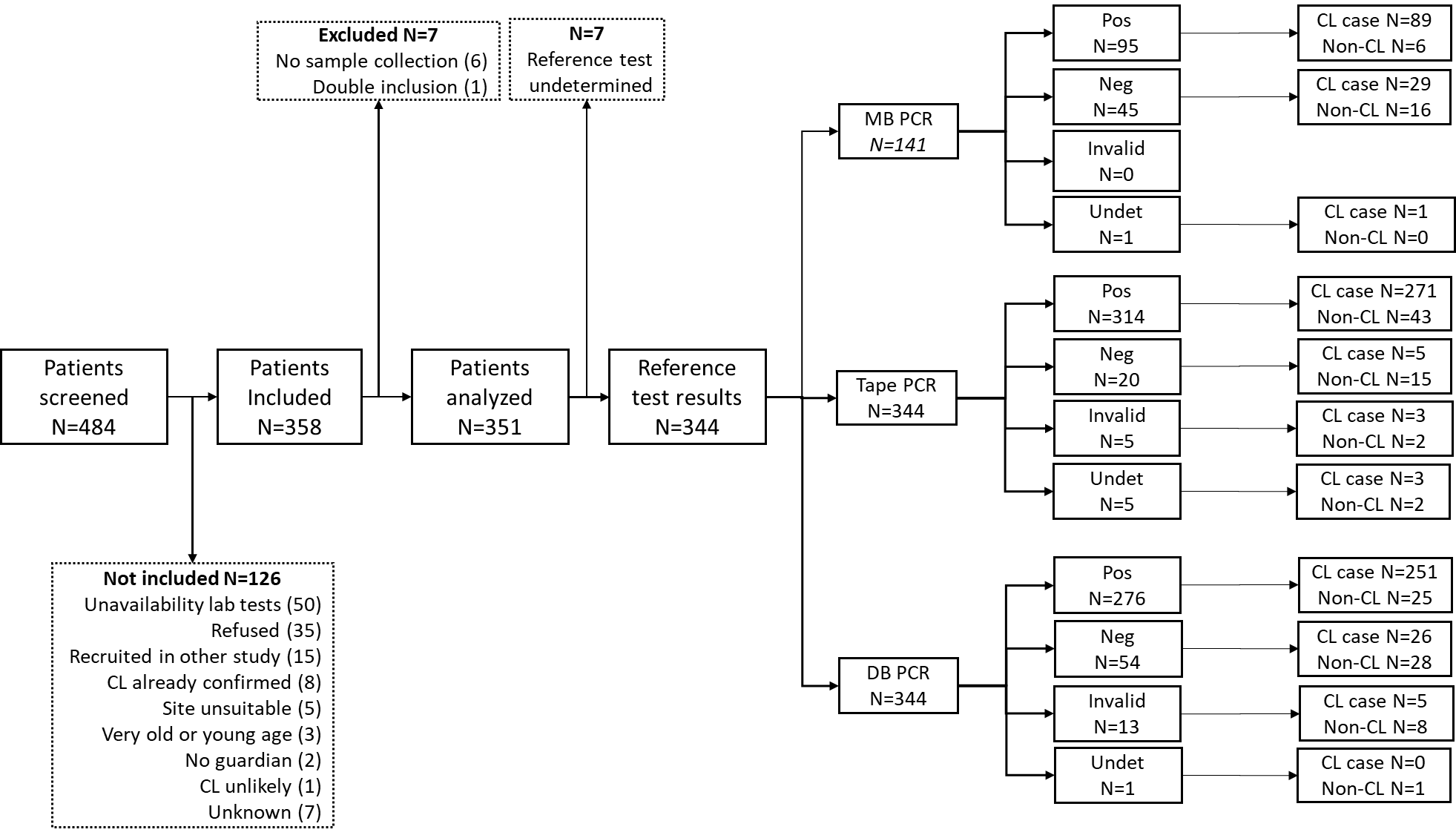

Supplement: ofae113_Supplementary_Data [file ofae113_supplementary_data.zip › 13. Supplementary Figure 2_updated.docx]
