# Supplementary material for: Evaluation of Less Invasive Sampling Tools for the Diagnosis of Cutaneous Leishmaniasis
Source: Open Forum Infect Dis. 2024 Feb 28;11(4):ofae113. doi: 10.1093/ofid/ofae113 (PMC10977625; doi:10.1093/ofid/ofae113)
Supplement: ofae113_Supplementary_Data [file ofae113_supplementary_data.zip › 14. Supplementary Figure 3.docx]

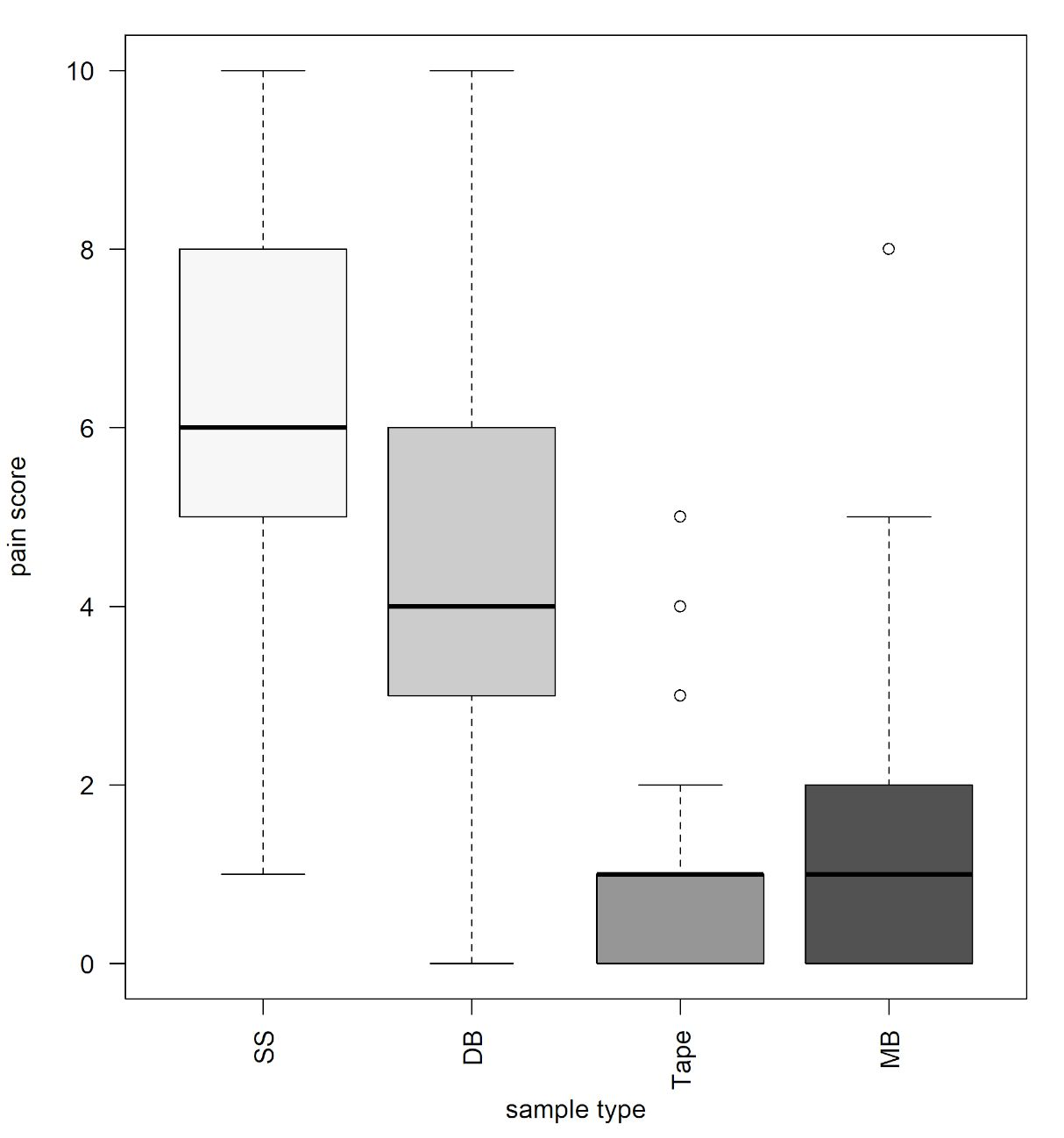


***Supplementary Figure 3. Pain scores from 0-10 for each sample type****. SS: skin slit, DB: dental broach, MB: Harpera microbiopsy.*
