# Supplementary material for: Evaluation of Less Invasive Sampling Tools for the Diagnosis of Cutaneous Leishmaniasis
Source: Open Forum Infect Dis. 2024 Feb 28;11(4):ofae113. doi: 10.1093/ofid/ofae113 (PMC10977625; doi:10.1093/ofid/ofae113)
Supplement: ofae113_Supplementary_Data [file ofae113_supplementary_data.zip › 15. Supplementary Figure 4.docx]

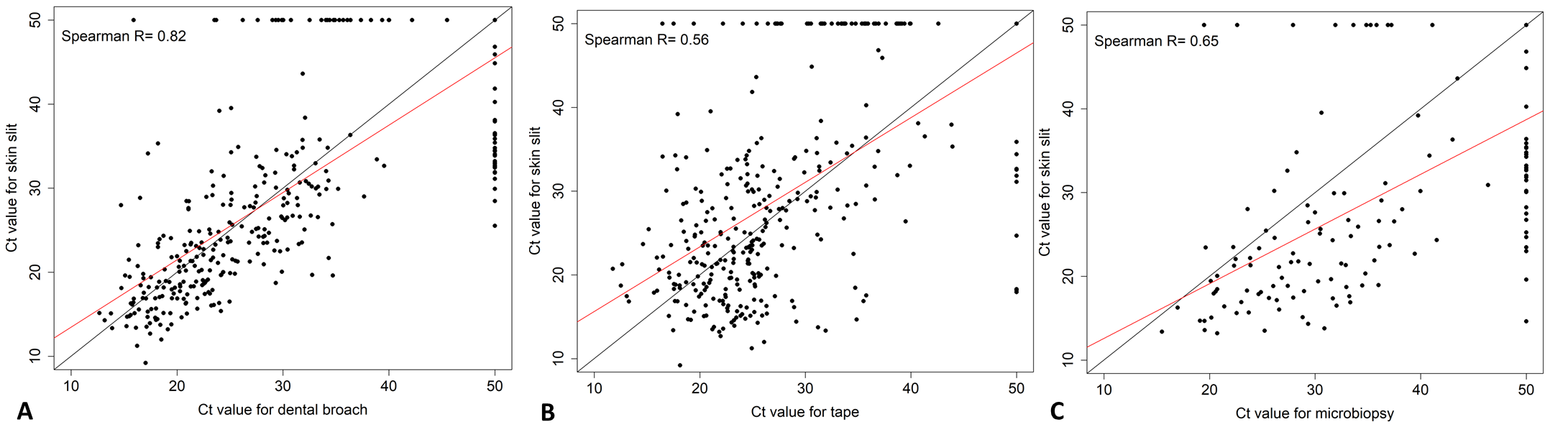
**Supplementary Figure 4. Scatterplots of the Ct-values of the different sample types compared to skin slit** Comparison of Ct-values between the skin slit and dental broach (A), skin slit and tape disc (B) and skin slit and microbiopsy (C). The red line represents the best fit (linear model), the black line represents the line of equality. Negative, invalid and undetermined samples were imputed with a Ct-value of 50.
