# Supplementary material for: Evaluation of Less Invasive Sampling Tools for the Diagnosis of Cutaneous Leishmaniasis
Source: Open Forum Infect Dis. 2024 Feb 28;11(4):ofae113. doi: 10.1093/ofid/ofae113 (PMC10977625; doi:10.1093/ofid/ofae113)
Supplement: ofae113_Supplementary_Data [file ofae113_supplementary_data.zip › 2. Supplementary Table 2_patient characteristics.docx]

| **Characteristic** | **Total N=351^a^** |
| --- | --- |
|  | **n (%)** |
| Male sex | 226 (64.4) |
| Age (years), median (IQR) | 24.0 (18.0 – 42.0) |
| Occupation (n=348) |  |
| Student | 133 (38.2) |
| Farmer | 84 (24.1) |
| Government employer | 36 (10.3) |
| Housewife | 36 (10.3) |
| Other | 61 (17.0) |
| Previous CL (n=350) | 36 (10.3) |
| Use of prior traditional treatment | 179 (51.0) |
| Lesion duration (months), median (IQR) | 9.0 (5.0 – 14.0) |
| Number of lesions (n=349) |  |
| 1 | 252 (72.2) |
| 2 | 44 (12.6) |
| 3 | 18 (5.2) |
| 4+ | 35 (10.0) |
| Size of lesion^b^ (cm), median (IQR) (n=346) | 4.8 (3.0 – 8.0) |
| Index lesion on face (n=349) | 310 (88.8) |
| Type of CL (n=349) |  |
| LCL | 205 (58.7) |
| MCL | 120 (34.4) |
| DCL | 24 (6.9) |
| Presentation of index lesion^c^ (n=349) |  |
| Crusted | 215 (61.6) |
| Swollen | 172 (49.3) |
| Erythema | 181 (51.9) |
| Plaque | 199 (57.0) |
| Ulcerated | 128 (36.7) |
| Papular | 69 (19.8) |
| Hyperpigmented | 86 (24.5) |
| Nodular | 62 (17.8) |
| Scaly | 52 (14.9) |

**Supplementary Table 2. Patient characteristics**

CL: cutaneous leishmaniasis; IQR: interquartile range; LCL: localized cutaneous leishmaniasis; MCL: muco-cutaneous leishmaniasis; DCL: diffuse cutaneous leishmaniasis

^a^If information was not available for all patients, the denominator is shown as (n=x)

^b^Measured on largest diameter of index lesion

^c^Lesions could have multiple presentations, therefore the sum of the different categories is larger than 351
