# Supplementary material for: Evaluation of Less Invasive Sampling Tools for the Diagnosis of Cutaneous Leishmaniasis
Source: Open Forum Infect Dis. 2024 Feb 28;11(4):ofae113. doi: 10.1093/ofid/ofae113 (PMC10977625; doi:10.1093/ofid/ofae113)
Supplement: ofae113_Supplementary_Data [file ofae113_supplementary_data.zip › 3. Supplementary Table 3_sens analysis excluding invalids.docx]

|  | **Cases^a^** | | **Non-cases^b^** | | **Diagnostic accuracy** | | | |
| --- | --- | --- | --- | --- | --- | --- | --- | --- |
| **Test** | **Pos** | **Neg** | **Pos** | **Neg** | **Sens (95%CI)** | **Spec (95%CI)** | **PPV (95%CI)** | **NPV (95%CI)** |
| **DB (n=323)** | 251 | 26 | 20 | 26 | 90.6 (86.6 – 93.5) | 56.5 (42.2 – 69.8) | 92.6 (88.9 – 95.2) | 50.0 (36.9 – 63.1) |
| **Tape (n=325)** | 271 | 5 | 34 | 15 | 98.2 (95.8 – 99.2) | 30.6 (19.5 – 44.5) | 88.9 (84.8 – 91.9) | 75.0 (53.1 – 88.1) |
| **MB *n=118** | 89 | 29 | 6 | 15 | 75.4 (66.9 – 82.3) | 71.4 (50.0 – 86.2) | 93.7 (86.9- 97.1) | 34.1 (21.9 – 48.9) |
| DB: dental broach PCR, MB: microbiopsy PCR; Tape: Tape PCR; Sens: sensitivity, Spec: specificity, PPV: positive predictive value, NPV: negative predictive value. Because the invalids and undetermined results are excluded here, the number of cases and non-cases is different for each index test analyzed. ^a^The CL cases are 277 for dental broach PCR, 276 for tape disc PCR and 118 for microbiopsy PCR. ^b^The non-CL cases are 46 for dental broach PCR, 49 for tape disc PCR and 21 for microbiopsy PCR. | | | | | | | | |

**Supplementary Table 3. Diagnostic accuracy when excluding invalid results from the index and reference tests using skin slit PCR as a reference test.**
