# Supplementary material for: Evaluation of Less Invasive Sampling Tools for the Diagnosis of Cutaneous Leishmaniasis
Source: Open Forum Infect Dis. 2024 Feb 28;11(4):ofae113. doi: 10.1093/ofid/ofae113 (PMC10977625; doi:10.1093/ofid/ofae113)
Supplement: ofae113_Supplementary_Data [file ofae113_supplementary_data.zip › 4. Supplementary Table 4_alternative reference tests.docx]

|  | **Skin slit PCR**  **N=344** | **Composite reference**  **N=351** | **Microscopy**  **N=351** |
| --- | --- | --- | --- |
| **SENSITIVITY** | | | |
|  | N=282 | N=307 | N=168 |
| **Skin slit** |  | 90.6 (86.8 – 93.3) | 98.2 (94.9 – 99.4) |
| **Dental broach** | 89.0 (84.8 – 92.1) | 90.9 (87.1 – 93.6) | 99.4 (96.7 – 99.9) |
| **Tape** | 96.1 (93.2 – 97.8) | 97.4 (94.9 – 98.7) | 98.2 (94.9 – 99.4) |
| **Microbiopsy^a^** | 74.8 (66.3 – 81.7) | 75.4 (67.3 – 82.0) | 90.3 (81.3 – 95.2) |
| **SPECIFICITY** | | | |
|  | N=62 | N=44 | N=183 |
| **Skin slit** |  | 86.4 (73.3 – 93.6) | 33.9 (27.4 – 41.0) |
| **Dental broach** | 58.1 (45.7 – 69.5) | 93.2 (81.8 – 97.7) | 37.2 (30.5 – 44.4) |
| **Tape** | 27.4 (17.9 – 39.6) | 45.5 (31.7 – 59.9) | 13.1 (9.0 – 18.8) |
| **Microbiopsy** | 72.7 (51.8 – 86.8) | 88.9 (67.2- 96.9) | 53.9 (42.8 – 64.7) |
| ^a^Since microbiopsy was introduced in the study later on (August 2021) results are only on a subset of patients. | | | |

**Supplementary Table 4. Sensitivity and specificity estimates when using different reference tests**
