# Supplementary material for: Evaluation of Less Invasive Sampling Tools for the Diagnosis of Cutaneous Leishmaniasis
Source: Open Forum Infect Dis. 2024 Feb 28;11(4):ofae113. doi: 10.1093/ofid/ofae113 (PMC10977625; doi:10.1093/ofid/ofae113)
Supplement: ofae113_Supplementary_Data [file ofae113_supplementary_data.zip › 5. Supplementary Table 5_incorporation bias.docx]

|  | **Cases^a^** | | **Non-cases^b^** | | **Diagnostic accuracy** | | | |
| --- | --- | --- | --- | --- | --- | --- | --- | --- |
| **Test** | **Pos** | **Neg** | **Pos** | **Neg** | **Sens (95%CI)** | **Spec (95%CI)** | **PPV (95%CI)** | **NPV (95%CI)** |
| **SS** | 216 | 7 | 38 | 83 | 94.7 (91.0 – 97.0) | 67.5 (58.8 – 75.1) | 85.0 (80.1 – 88.9) | 92.2 (84.8 – 96.2) |
| **DB** | 219 | 9 | 38 | 84 | 96.1 (92.7 – 97.9) | 68.3 (59.6 – 85.9) | 85.2 (80.4 – 89.0) | 90.3 (82.6 – 94.8) |
| **Tape** | 201 | 41 | 11 | 93 | 82.0 (76.8 – 86.3) | 87.7 (80.1 – 92.7) | 94.8 (90.9 – 97.1) | 69.4 (61.2 – 76.6) |
| **MB** | 74 | 33 | 1 | 39 | 68.5 (59.3 – 76.5) | 97.5 (87.1 – 99.6) | 98.7 (92.8 – 99.8) | 54.2 (42.7 – 65.2) |
| DB: dental broach PCR, MB: microbiopsy PCR; Tape: Tape PCR; Sens: sensitivity, Spec: specificity, PPV: positive predictive value, NPV: negative predictive value. Because the composite reference test is different for every index test, the number of cases and non-cases is different for each index test analyzed. ^a^The CL cases are 228 for DB and SS, 245 for tape disc PCR and 108 for microbiopsy PCR. ^b^The non-CL cases are 123 for SS and DB, 106 for tape and 40 for microbiopsy PCR. | | | | | | | | |

**Supplementary Table 5. Diagnostic accuracy using a composite reference based on cutoffs without including the index test**
